# Performance of Vision-Enabled Large Language Models in Image-Based Electrocardiogram Interpretation: Exploratory Evaluation

**DOI:** 10.2196/86692

**Published:** 2026-06-03

**Authors:** Nibras Soubh, Eva Rasenack, Helge Haarmann, Felix Wiedmann, Markus Zabel, Constanze Schmidt, Rayan Suliman, Leonard Bergau

**Affiliations:** 1 Department of Cardiology and Pneumology, University Medical Center Göttingen (UMG), Robert-Koch-Str. 40, Göttingen, 37075, Germany, 49 15114609645; 2 German Centre for Cardiovascular Research, Partner Site Lower Saxony, University of Göttingen, Göttingen, Germany; 3 Institute of Clinical Chemistry and Laboratory Medicine, Municipal Hospital Dresden, Dresden, Germany

**Keywords:** artificial intelligence, AI, electrocardiography, ECG, machine learning, large language models, LLMs

## Abstract

**Background:**

Vision-enabled large language models (VE-LLMs) have the potential to provide flexible and explainable medical image interpretation. However, their real-world performance on clinical data, such as 12-lead electrocardiograms (ECGs), has not been systematically assessed.

**Objective:**

This study aimed to evaluate the diagnostic accuracy and reliability of state-of-the-art generalist VE-LLMs in interpreting real-world ECG images.

**Methods:**

We tested 6 generalist VE-LLMs (ChatGPT-5, ChatGPT-4, Gemini 2.5, Copilot, Claude Sonnet-4, and Claude Opus-4.1) using 70 deidentified ECG images. A standardized prompt requested 9 determinations: rhythm, first-degree atrioventricular (AV) block, intraventricular conduction block and pattern, corrected QT (QTc) prolongation, premature atrial and ventricular contractions, ischemic ST-segment deviation, and axis deviation. An expert consensus served as the reference standard. Moreover, 2 image-based ECG-specialized LLMs (PULSE-7B and ECG-Instruct-Llama-3.2-11B-Vision) were tested for exploratory comparison. Model outputs were evaluated using overall and per-category diagnostic metrics.

**Results:**

Overall balanced accuracy across generalist models ranged from 50.1% to 61.8% (Cochran Q, *P*<.001). ChatGPT-5 achieved the highest balanced accuracy (61.8%) but had the slowest response time (median 276, IQR 110-407 s), whereas Copilot responded within a median of 3 (IQR 2-4) seconds. Balanced accuracy for rhythm classification ranged from 38.6% to 55.8%, but sensitivity for atrial fibrillation among generalist models was ≤11.1%, detecting either none or only 1 of the 9 cases. Detection of first-degree AV block (sensitivity 0%‐22%; 0/9 to 2/9) and QTc prolongation (sensitivity 0%‐45.5%; 0/22 to 10/22) was poor. Intraventricular block was identified with up to 67.8% balanced accuracy, but correct subtype assignment was ≤44% (≤11/25). ST-segment deviation sensitivity was <25% for all generalist models (highest 3/14). Agreement with expert interpretation was low, with Cohen κ indicating poor-to-fair concordance (κ≤0.39). Specialized models achieved overall balanced accuracy of 56.5% (ECG-Instruct-Llama-3.2-11B-Vision) and 64.4% (PULSE-7B), with PULSE-7B showing higher task-specific balanced accuracy in rhythm classification and ectopic beats detection (up to 86.3% and 89.2%, respectively).

**Conclusions:**

VE-LLMs showed moderate overall performance but mostly low sensitivity and limited agreement with expert ECG interpretation. Current performance remains inconsistent across models and diagnostic categories and is insufficient to support clinical deployment.

## Introduction

Large language models (LLMs) are artificial intelligence (AI) systems trained on vast text corpora to generate human-like responses. The public release of the first generalist LLM—OpenAI’s ChatGPT—in late 2022 marked a major inflection point. In just 2 years, LLMs have influenced many aspects of daily life and professional practice, with successive generations steadily expanding conversational and problem-solving capabilities. These models are now being explored across education, law, and scientific research, reshaping workflows while raising new societal and ethical questions.

In medicine, LLMs have shown notable promise on knowledge-based tasks. For example, Google’s Med-PaLM and Med-PaLM 2 achieved scores exceeding the passing threshold for the USMLE (United States Medical Licensing Examination) [1]. Meanwhile, multiple studies evaluating publicly accessible LLMs across international medical examinations indicate that newer versions can perform well, although results vary by model, language, and examination difficulty [2,3]. Beyond examinations, LLMs are being piloted for clinical use cases, such as drafting documentation, summarizing literature, and answering patient queries in lay terms [4,5]. However, clinical deployment requires rigorous validation and appropriate regulatory frameworks, particularly for data protection and patient privacy.

The 12-lead electrocardiogram (ECG) is a cornerstone diagnostic tool in cardiology and emergency medicine. Recent advances in AI—especially deep learning—have yielded strong results in automated ECG analysis. Convolutional neural network (CNN) models trained on large ECG datasets can achieve expert-level interpretation and may uncover latent features not apparent to human readers. For instance, multiple AI ECG models have been shown to predict the near-term onset of atrial fibrillation (AF) from ECGs recorded in apparent sinus rhythm (SR), detecting subclinical signatures invisible to clinicians [6,7]. EchoNext—a deep learning model trained on more than one million ECG waveforms and imaging records—outperformed cardiologists in detecting structural heart disease across diverse clinical settings and patient groups [8].

This emerging field of AI-enhanced ECG analysis promises faster and potentially more sensitive screening for cardiac pathology using a ubiquitous, inexpensive test. Despite progress, most AI ECG solutions remain task specific, have limited accessibility, require waveform (signal data) as input, and lack the flexible reasoning of human interpreters. Integrating advanced LLMs into image-based ECG interpretation could combine the pattern recognition strengths of deep learning with the linguistic and contextual reasoning of LLMs. Historically, generalist LLMs accepted only text input. Newer models are multimodal, accepting images in addition to text. GPT-4 with “Vision” (released in 2023) demonstrated that an LLM augmented with a visual encoder can describe images and answer questions about them. Competing vision-enabled LLMs (VE-LLMs) soon followed. Early experience showed impressive performance on general images—object recognition, chart interpretation, handwriting—yet the ability of generalist VE-LLMs to interpret complex medical images remains uncertain, with only a few studies assessing earlier versions on simplified, textbook ECG test sets [9,10].

Previous studies evaluating ChatGPT-4 have largely focused on single-model assessments, limited diagnostic tasks, or simplified test settings, often without structured benchmarking across multiple models and clinically derived datasets. Given the rapid evolution of LLM capabilities, we conducted a comprehensive evaluation of the newest VE-LLMs as of August 2025 (including ChatGPT-5 and Claude Opus-4.1) on a clinically relevant image understanding task: 12-lead ECG interpretation. We focused on fundamental ECG findings—rhythm, atrioventricular (AV) and intraventricular conduction delays (IVCDs), ectopy, and ischemia—routinely assessed by clinicians and automated algorithms. By comparing 6 prominent generalist models side-by-side, we sought to determine how performance varies across models and diagnostic categories and how models compare with human experts and with 2 further ECG-specialized image-based LLMs (PULSE-7B and ECG-Instruct-Llama-3.2-11B-Vision).

## Methods

### Ethical Considerations

This retrospective diagnostic study was conducted at the University Medical Center Göttingen, Germany, using deidentified 12-lead ECG images interpreted by AI models, with expert human interpretation as the reference standard. The study was reviewed and approved by the local institutional ethics committee (approval 18/08/24). In accordance with the ethics committee decision and given the retrospective design and the use of fully anonymized data, the requirement for informed consent was waived. All data were handled in accordance with institutional data protection policies, and no identifiable personal information was included in the dataset or study outputs. No participants received financial compensation.

### ECG Data—Acquisition and Preparation

Seventy 12-lead ECGs were collected from consecutive patients admitted to the heart rhythm ward between June 1 and 15, 2025. Recordings were obtained using the standard hospital device (Cardiovit AT-102 G2; Schiller Medizintechnik GmbH) at 500 Hz, with a paper speed of 50 mm/s and 10 mm/mV calibration. Each ECG was originally in PDF format and included patient metadata and automated interpretations. Patient information, demographics, and machine-generated measurements were removed using Inkscape (Inkscape Project, version 1.3.2), leaving only the waveform grid and tracings. Images were exported as high-resolution JPEG files (300 dots per inch). The final dataset contained 70 ECG images, each a single page with the standard 12 leads (I, II, III, aVR, aVL, aVF, and V1-V6). Two physicians independently reviewed all ECGs to determine the correct findings for each of the 9 diagnostic questions (detailed in the following section). Discrepancies were resolved by consensus discussion. These expert interpretations served as the reference standard. Interval assessments (PR, QRS, and QTc) by expert readers were performed using manual measurements from the ECG images based on standard calibration. Difficulty of ECGs was classified into three categories: (1) easy: normal ECG or isolated clear pathological findings with no baseline disturbances or borderline measurements; (2) intermediate: borderline measurements, subtle findings, baseline artifacts, and the co-occurrence of multiple pathological findings; and (3) difficult: multiple or rare pathological findings and multiple borderline measurements.

### Selection and Inference of LLM Models

We identified 7 publicly available generalist multimodal LLMs (accepting image and text input) that represented the top-tier models from major AI developers: ChatGPT-4 and ChatGPT-5 (OpenAI), Gemini 2.5 (Google), Copilot (Microsoft; GPT-4-based, with “Think Deeper” activated), Grok-4 (xAI), Claude Sonnet-4, and Claude Opus-4.1 (Anthropic).

We excluded Meta AI’s LLM, as no publicly available multimodal model was provided as of August 2025, and DeepSeek because its vision-enabled chat model is limited to text extraction from images as stated by the model itself. Grok-4 inference was performed via the Perplexity Pro platform; however, it was excluded from final analysis following the disclosure of a “silent fallback bug” by the platform provider. Due to this technical issue, queries intended for Grok-4 were intermittently routed to ChatGPT-4 without user notification, confounding the model-specific results. All other models were inferred via their public web interfaces using default settings and within their usage policies and rate limits. As inference was conducted through publicly accessible web interfaces rather than APIs, exact backend model version identifiers were not exposed to users. Model provenance was therefore documented using platform name, model designation, subscription level, geographic inference location, client environment, and precise inference dates (Table S1 in Multimedia Appendix 1), consistent with current reporting practices for evaluations of publicly deployed multimodal systems.

Additionally, we evaluated 2 open-source LLMs specialized for ECG image interpretation: ECG-Instruct-Llama-3.2-11B-Vision (ECG-Instruct-Llama-3.2), a multimodal Llama-3.2 model adapted to ECGs using parameter-efficient Low-Rank Adaptation (LoRA) fine-tuning [11]; and PULSE-7B, a smaller LLaVA-based vision language model instruction tuned on ECG image–text data [12]. Model inference was performed in a cloud-based high-performance computing environment (Google Colab Pro+, Google LLC), with detailed environment and inference settings provided in Table S2 in Multimedia Appendix 1.

### Prompt Design and Results Mapping

Pilot testing was performed on 20 ECGs (not included in the study’s dataset). Short prompts often produced incomplete or pattern-based answers. To mitigate this, we optimized a prompt to explicitly instruct step-by-step visual analysis. We incorporated advice from the models themselves and known best practices for eliciting analytical responses. Broadly applicable models’ suggestions (eg, emphasizing lead-by-lead analysis and the explicit use of visual detail) were incorporated, while the final standardized prompt was defined by the investigators and applied uniformly across models. The final prompt given to all models for each ECG was as follows:

“Please perform a structured, lead-by-lead visual analysis of this ECG image (paper speed: 50 mm/s, amplitude: 10 mm/mV). For each lead (I, II, III, aVR, aVL, aVF, V1–V6), identify the P, QRS, T waves and describe their durations and characteristics. Do not rely on pattern-matching shortcuts or global assumptions – use the detailed visual information from the image. After analyzing, answer the following numbered questions (with a single answer each):

1. What is the cardiac rhythm? (Choose one: “sinus rhythm,” “atrial fibrillation,” “paced rhythm,” or “other”)
2. Is there a first-degree AV block? (“yes” or “no”)
3. Is an intraventricular conduction block present? (“yes” or “no”)
4. If yes, which type of bundle branch block is it? (Choose “RBBB,” “LBBB,” or “nonspecific”)
5. Is the QTc interval >450 ms (QT prolongation)? (“yes” or “no”)
6. Are there any premature supraventricular beats (PACs)? (“yes” or “no”)
7. Are there any premature ventricular beats (PVCs)? (“yes” or “no”)
8. Are there significant ST-segment deviations suggestive of myocardial ischemia? (“yes” or “no”)
9. Is the QRS axis abnormal (deviated beyond +90° or –30°)? (“yes” or “no”).”

We explicitly mentioned calibration and discouraged shortcuts to encourage actual measurement. We also constrained answers to the required format (yes or no or single option) for easier evaluation. All models received the exact same prompt text with no vendor-specific adjustments to default system settings. If a model’s answer did not follow the format (eg, gave explanations or multiple options), we recorded its final explicit answer for each numbered item. No further prompt engineering or follow-up questions were applied. Each model thus output 9 answers per ECG, which we collected for analysis. Authors extracting and mapping model outputs were blinded to the correct answers. Additionally, we measured the response latency for each generalist model: specifically, the time from submitting the prompt to the model beginning to output the first word of its answer.

Given their substantially smaller size and domain-specific optimization compared with frontier generalist multimodal LLMs—which are typically optimized for complex, multistep prompting—we additionally evaluated the ECG-specialized models using a short “impression-style” prompt to provide a model-appropriate testing condition. The short prompt was as follows:

“Analyze this 12-lead ECG and provide a concise cardiology-style impression (rhythm, conduction, axis, QTc, ectopy, ischemia)”

Generalist LLMs and the standard-prompted PULSE-7B adhered to the structured prompt and usually produced numbered answers in the requested format without contradictory or missing responses, although isolated deviations from the predefined option set required minimal manual mapping during dataset construction (Multimedia Appendix 2). The ECG-Instruct-Llama-3.2 model did not consistently follow the structured response format and frequently generated narrative ECG reports instead of discrete answers. Therefore, a postprocessing step was applied for this model and for the short-prompted PULSE-7B. Model outputs were manually reviewed and mapped to predefined diagnostic categories. A category was recorded as positive only when a corresponding finding (eg, bundle branch block and QT prolongation) was stated explicitly and unequivocally. If a finding was not mentioned or described ambiguously, it was classified as negative for that category.

### Outcome Measures

The reference answers were (1) rhythm classified as SR, AF, paced, or other; (2) first-degree AV block present or not (defined as PR interval >200 ms); (3) intraventricular conduction block present or not (defined as QRS prolongation >120 ms); (4) if an intraventricular conduction block present, type classified as right bundle branch block, left bundle branch block, or nonspecific IVCD. Performance metrics in this category were calculated as a multiclass classification across the full dataset (correct or incorrect), with “no block” treated as a separate class; (5) QT prolongation defined as corrected QT (QTc) >450 ms (using Bazett’s formula; a single threshold was applied as patient sex information was not available); (6) premature atrial contractions (PACs) present or not; (7) premature ventricular contractions (PVCs) present or not; (8) ST-segment changes significant for ischemia or not, measured at the J-point in contiguous leads, with a cutoff for depression of ≥2 mm and elevation of ≥1 mm in all leads except V2 and V3, where a cutoff of ≥2 mm was applied due to unavailability of age and sex data; in the presence of bundle branch block or paced rhythm, modified Sgarbossa criteria were used; and (9) QRS axis abnormal or not (right axis >+90° or left axis <–30°).

### Performance Metrics and Statistics

Given the exploratory nature of this study, no prospective power analysis was performed. A post-hoc calculation of the minimum detectable difference for the primary outcome indicated that with a sample size of 70, the study was powered (80%; α=.05) to detect differences of at least 22% in the overall accuracy. We calculated conventional classification metrics for each model on each question: accuracy, sensitivity, specificity, positive predictive value, negative predictive value, balanced accuracy, and *F*_1_-score. These were computed with the binary interpretation (eg, for QT prolongation: “yes”=positive and “no”=negative). For multiclass categories, we reported overall accuracy. Ninety-five percent CIs were calculated using the Wilson method for proportions and bootstrap resampling where appropriate. To contextualize model performance relative to a naive baseline, we additionally compared each model with a majority class classifier (ZeroR). The Cochran Q test was used to investigate overall differences in accuracy among the models. Paired comparisons between each model and the majority class classifier were performed using the McNemar test with Bonferroni correction, and the adjusted *P* values were reported. To assess agreement between each model’s classifications and the expert reference, Cohen κ was used. κ values were interpreted as <0.20 (poor), 0.21 to 0.40 (fair), 0.41 to 0.60 (moderate), 0.61 to 0.80 (good), and >0.80 (excellent agreement beyond chance). We also calculated the per-case total score for each model (out of 9 questions per ECG) as a crude measure of overall ECG interpretation performance. The median scores were compared between models. Finally, response times were summarized per model (median, IQR) as a measure of efficiency. For continuous or ordinal outcomes such as the per-case score and response time, we used Friedman ANOVA to test for global differences and Wilcoxon signed-rank tests for pairwise model comparisons. All hypothesis tests were conducted using a 2-sided approach, with statistical significance defined as *P*<.05. Statistical analysis was performed using R (R Foundation for Statistical Computing, version 4.4.0); the analysis codes are provided in Multimedia Appendix 3.

## Results

### Dataset Characteristics

The 70 ECGs encompassed a wide spectrum of findings. Normal SR was present in 57 (81%) cases, AF in 9 (13%) cases, pacemaker rhythm in 3 (4%) cases, and AF with pacemaker stimulation in 1 (1%) case. First-degree AV block was present in 9 (13%) cases. Intraventricular conduction block was present in 25 (36%) cases–including 9 with right bundle branch block, 8 with left bundle branch block, and 8 with nonspecific IVCD. QT prolongation was noted in 22 cases (31%). PACs were rare (n=3, 4%), whereas PVCs were seen in 10 (14%) cases. ST-segment changes suggestive of myocardial ischemia were present in 14 cases (20%). Abnormal QRS axis (right or left axis deviation) was present in 12 cases (17%). The anonymized ground-truth label matrix is provided in Multimedia Appendix 4. The interrater agreement between the 2 cardiologists was high, with an overall percent agreement of 97.3%. Category-specific agreement ranged from 94.2% (suspected ST-segment changes) to 100% (rhythm and PVCs). Cohen κ indicated substantial to almost perfect reliability across domains, ranging from 0.80 for suspected ST-segment changes to 1.00 for rhythm and PVCs classification. Detailed interrater agreement metrics are provided in Tables S3 in Multimedia Appendix 5.

### Model Response Times

**Figure 1.**
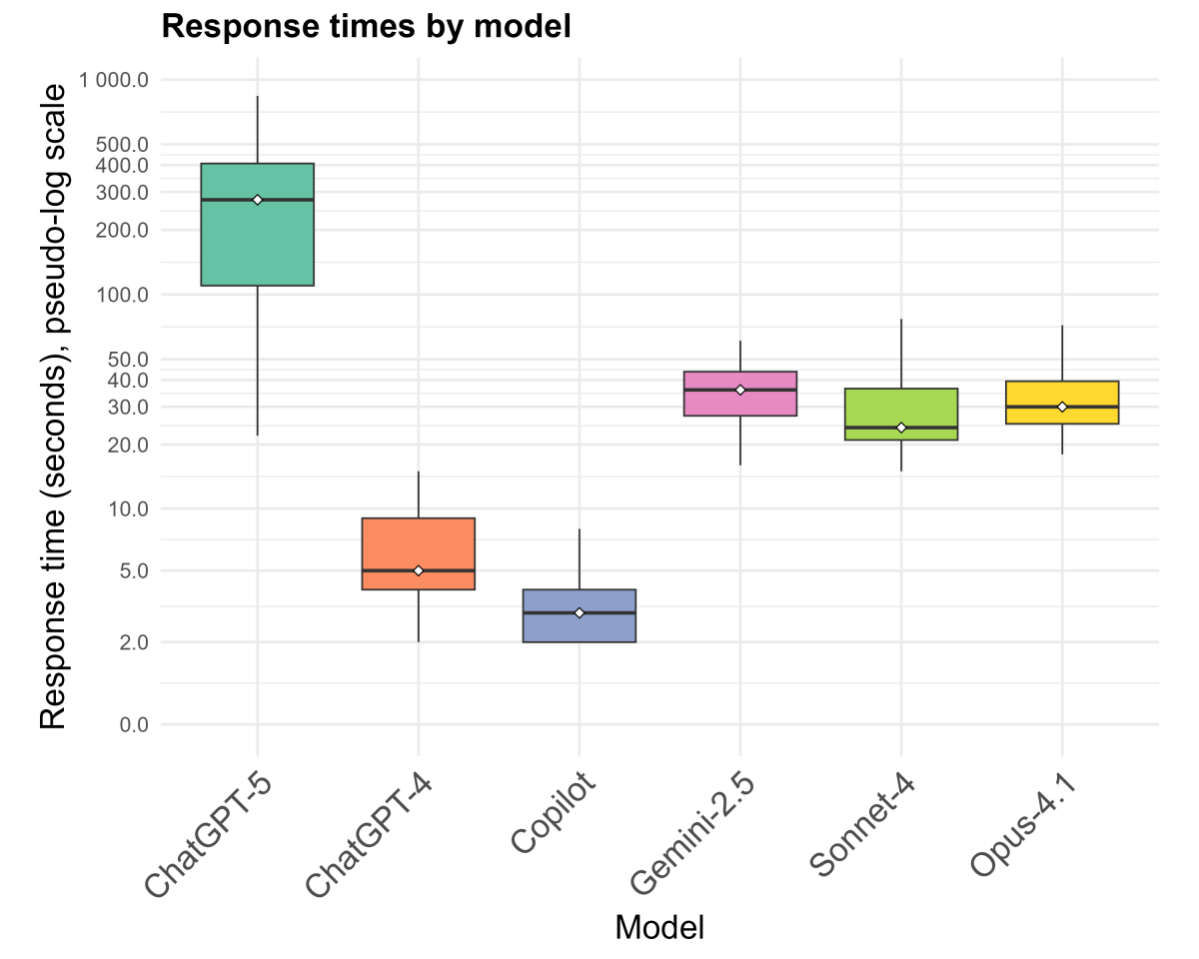
Response times. Response times of generalist vision-enabled large language models (LLMs) when interpreting 12-lead electrocardiogram (ECG) images from a retrospective dataset of 70 deidentified ECGs collected during routine clinical care in a cardiology ward at the University Medical Center Göttingen, Germany. Model inference was conducted in July to August 2025. The figure shows boxplots of the time each model required to begin generating an output.

User-perceived response latency varied strikingly across models (Figure 1). Microsoft Copilot was the fastest, with a median response time of 3 (IQR 2-4) seconds. ChatGPT-4 was also relatively quick (median 5, IQR 4‐9 s). Anthropic’s models were slower: Claude Sonnet-4 with a median of 24 (IQR 15‐36) seconds, and Claude Opus-4.1 with a median of 30 (IQR 25‐40) seconds. Gemini 2.5 responded in a median of 36 (IQR 27‐44) seconds. The slowest by far was ChatGPT-5, with a median latency of 276 seconds (4.6 min) and very high variability (IQR 110‐407 s). In 1 case, it took 14 minutes to begin responding. The difference spanned nearly 2 orders of magnitude, from seconds to minutes. Statistical testing confirmed a significant overall difference (Friedman *χ²*_5_=299.5; *P<*.001; Kendall W=0.855). Pairwise comparisons showed ChatGPT-5 was significantly slower than every other model (adjusted *P*<.001), and among the rest, Copilot was significantly faster than all other models (adjusted *P*<.001).

### Overall Accuracy

**Figure 2.**
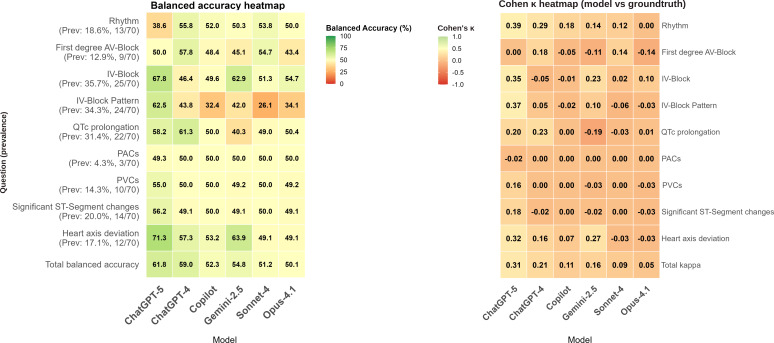
Heatmaps of balanced accuracy and Cohen κ. Balanced accuracy (left) and Cohen κ for agreement with expert interpretation (right) across electrocardiogram (ECG) interpretation tasks, shown as heatmaps for all generalist vision-enabled large language models. The models were evaluated using a retrospective dataset of 70 deidentified 12-lead ECGs collected during routine clinical care in a cardiology ward at the University Medical Center Göttingen, Germany, with expert consensus as the reference standard; model inference was conducted in July to August 2025. Balanced accuracy values are presented as percentages, and both balanced accuracy and κ are visualized using a color scale (green=higher values and orange or red=lower values). IV-Block: intraventricular block; IV-Block Pattern: intraventricular block pattern; QTc: corrected QT interval; PAC: premature atrial contraction; PVC: premature ventricular contraction.

The overall accuracy was estimated across all 9 diagnostic questions (computed as total correct answers out of 630 possible [70 ECGs×9 questions]). ChatGPT-5 achieved the highest aggregate accuracy (correct: 493/630, 78.3%) among generalist models, ahead of Claude Opus-4.1 (470/630, 74.6%) and Claude Sonnet-4 (464/630, 73.7%). Copilot was fourth (452/630, 71.7%), followed by Gemini 2.5 (445/630, 70.6%) and ChatGPT-4 (438/630, 69.5%). The range between the highest and lowest accuracy was relatively wide (approximately 10 percentage points), and the Cochran Q test on the full 6×630 binary outcome matrix was statistically significant (*P<*.001). After correction for category imbalances, ChatGPT-5 also achieved the highest overall balanced accuracy (61.8%) and *F*_1_-score (38%). Other models showed progressively lower balanced accuracy and *F*_1_-scores reaching 50.1% and 13%, respectively (Claude Opus-4.1), as shown in Table S4 in Multimedia Appendix 5. The ZeroR (majority class) classifier achieved 79% accuracy, higher than all other models. In McNemar testing with Bonferroni correction, a statistically significant asymmetry in discordant classifications favoring ZeroR was observed in all models except for ChatGPT-5 (Table S5 in Multimedia Appendix 5). This aggregate view masks considerable variation in performance by question type, which we detail below. Figure 2 provides an overview of each model’s overall and category-specific balanced accuracy and agreement with human experts across the 9 diagnostic questions. ZeroR exceeded model per-task accuracy for all tasks except: ChatGPT-5 (4/9 tasks where model >ZeroR) and ChatGPT-4 (1/9 task where model>ZeroR). Detailed overall performance metrics, corresponding 95% CIs, the heatmap of unbalanced accuracy, and the full ZeroR comparisons across models and task categories are provided in MultimediaAppendices 5  6.

### Models' Performance Across Difficulty Spectrum

**Figure 3.**
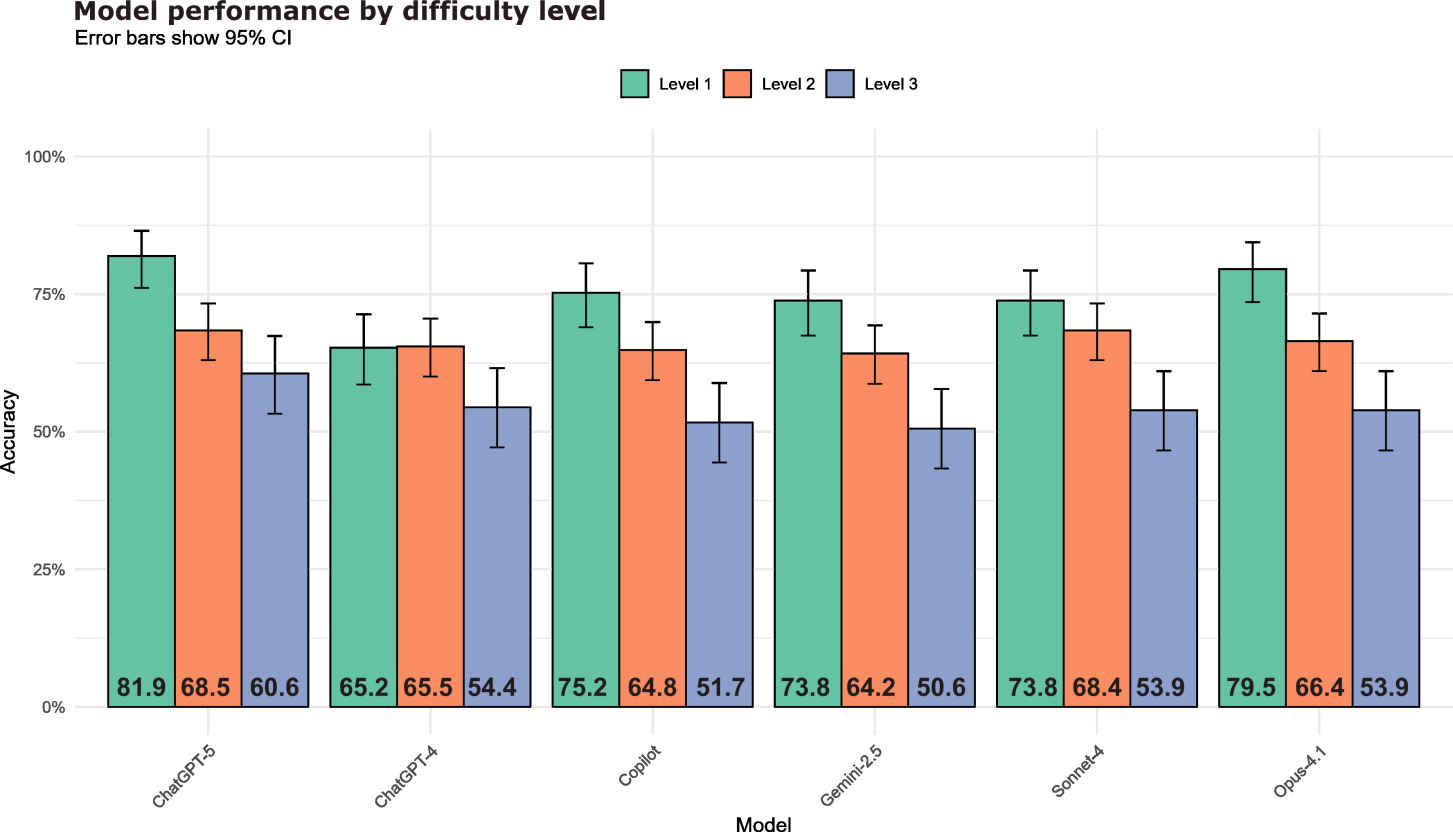
Accuracy of vision-enabled generalist large language models stratified by electrocardiogram (ECG) difficulty level (levels 1‐3), reported as the proportion of correct responses with 95% CIs (error bars). Models were evaluated using a retrospective dataset of 70 deidentified 12-lead ECGs collected during routine clinical care in a cardiology ward at the University Medical Center Göttingen, Germany, with expert consensus as the reference standard. Model inference was conducted in July to August 2025. Statistical comparisons were performed per model using 2-sided Pearson *χ*² tests on 3×2 contingency tables (correct vs incorrect responses) stratified by difficulty level.

The 70 ECGs were stratified by expert-rated difficulty into 21 (30%) level-1 (straightforward), 31 (44.3%) level-2 (intermediate), and 18 (25.7%) level-3 (challenging) tracings. A consistent and statistically significant decrease in diagnostic accuracy was observed as the complexity of the tracings increased. This pattern was monotonic for every individual model as shown in Figure 3. ChatGPT-5 achieved the highest metrics in the most challenging stratum, achieving an overall accuracy and balanced accuracy of 60.6% and 58.6%, respectively. Detailed overall accuracies, balanced accuracies, and *F*_1_-scores stratified by ECG difficulty are provided in Tables S6-7 in Multimedia Appendix 5.

### Rhythm Classification

Across all models, rhythm was correctly identified in 72.9% (n= 51/70) to 82.9% (n= 58) of cases. Statistical comparison showed a significant difference in the proportion of correct answers among models (the Cochran Q test, *P*<.001). The highest performance metrics among generalist models were achieved by Claude Sonnet-4 and ChatGPT-4 with the following accuracy, balanced accuracy, and agreement with human experts (κ): 82.9%, 53.8%, and 0.12 and 81.4%, 55.8%, and 0.29, respectively. All generalist models showed high sensitivity for identifying SR (89%‐100%) but very low sensitivity for AF (≤11.1%). Most generalist LLMs failed to detect any AF cases. Only Copilot and Claude Sonnet-4 detected 1 of 9 (sensitivity 11%). Despite higher accuracies, other adjusted diagnostic metrics, balanced accuracy and agreement with the human experts, were rather low and indicated poor performance as shown in Table 1.

**Table 1.** Measures regarding rhythm classification^a^.

Measure	OpenAI ChatGPT-4	OpenAI ChatGPT-5	Google Gemini 2.5	Microsoft Copilot	Claude Sonnet-4	Claude Opus-4.1
Diagnostic metric						
Accuracy, % (95% CI)	81.4 (70.8‐88.8)	72.9 (61.5‐81.9)	78.6 (67.6‐86.6)	81.4 (70.8‐88.8)	82.9 (72.4‐89.9)	81.4 (70.8‐88.8)
Balanced accuracy, % (95% CI)	55.8 (44.2‐70.6)	38.6 (33.3‐43.4)	50.3 (42.9‐61)	52 (45.3‐63.6)	53.8 (50‐62.5)	50 (50‐50)
Agreement measure						
Cohen κ^b^	0.29	0.39	0.14	0.18	0.12	0

a Performance of generalist vision-enabled large language models in classifying cardiac rhythm from 70 deidentified 12-lead electrocardiograms collected during routine clinical care in a cardiology ward at the University Medical Center Göttingen, Germany, with expert consensus as the reference standard. Model inference was conducted in July to August 2025.

b Agreement with expert interpretation measured using Cohen κ test.

### Identification of First-Degree AV Block

Accuracy for detecting first-degree AV block ranged from 75.7% (Claude Opus-4.1) to 87.1% (ChatGPT-5 and Claude Sonnet-4). However, these high accuracies primarily reflected a tendency of many models to answer “no” for all cases, thereby correctly classifying true negatives but missing all positives. Balanced accuracy dropped in all models with the lowest in Claude Opus-4.1 (43.4%) and the highest in ChatGPT-4 (57.8%). Agreement with human experts, assessed using Cohen κ, was generally poor, ranging from κ=–0.14 (Claude Opus-4.1) to κ=0.18 (ChatGPT-4). The Cochran Q test found significant overall difference among models for AV block detection (*P*<.001). Table 2 summarizes diagnostic metrics for this task.

**Table 2.** Measures regarding identification of first-degree atrioventricular block^a^.

Measures	OpenAI ChatGPT-4	OpenAI ChatGPT-5	Google Gemini 2.5	Microsoft Copilot	Claude Sonnet-4	Claude Opus-4.1
Diagnostic metric
Sensitivity, % (95% CI)	22.2 (6.3‐54.7)	0 (0‐29.9)	0 (0‐29.9)	0 (0‐29.9)	11.1 (2‐43.5)	0 (0‐29.9)
Specificity, % (95% CI)	93.4 (84.3‐97.4)	100 (94.1‐100)	90.2 (80.2‐95.4)	96.7 (88.8‐99.1)	98.4 (91.3‐99.7)	86.9 (76.2‐93.2)
PPV^b^, % (95% CI)	33.3 (9.7‐70)	NA^c^	0 (0‐39)	0 (0‐65.8)	50 (9.5‐90.5)	0 (0‐32.4)
NPV^d^, % (95% CI)	89.1 (79.1‐94.6)	87.1 (77.3‐93.1)	85.9 (75.4‐92.4)	86.8 (76.7‐92.9)	88.2 (78.5‐93.9)	85.5 (74.7‐92.2)
Accuracy, % (95% CI)	84.3 (74-91)	87.1 (77.3‐93.1)	78.6 (67.6‐86.6)	84.3 (74-91)	87.1 (77.3‐93.1)	75.7 (64.5‐84.2)
Balanced accuracy, % (95% CI)	57.8 (45.2‐74.2)	50 (50‐50)	45.1 (41.1‐48.4)	48.4 (45.8‐50)	54.7 (47.8‐67.7)	43.4 (38.9‐47.5)
*F*_1_-score (95% CI)	26.7 (11.8‐57.1)	NA	NA	NA	18.2 (12.5‐53.3)	NA
Agreement measure
κ^e^	0.18	0	−0.11	−0.05	0.14	**−0.14**

a Performance of generalist vision-enabled large language models in detecting first-degree atrioventricular block from 70 deidentified 12-lead electrocardiograms collected during routine clinical care in a cardiology ward at the University Medical Center Göttingen, Germany, with expert consensus as the reference standard. Model inference was conducted in July–August 2025. Prevalence of first-degree atrioventricular block was 9/70 (13%).

b PPV: positive predictive value.

c NA: not applicable because no positives and/or no positive predictions.

d NPV: negative predictive value.

e Agreement with expert interpretation measured using Cohen κ.

### Identification and Classification of Intraventricular Conduction Blocks

Model accuracy in detecting and classifying these wide QRS patterns varied significantly (Cochran Q, *P*<.001). ChatGPT-5 showed the highest performance metrics in this task, correctly identifying block in 15 cases and normal conduction in 34 cases (accuracy: 70%, balanced accuracy: 67.8%, *F*_1_-score: 58.8%, and agreement with human raters: *κ*=0.35). In contrast, ChatGPT-4 (accuracy: 35.7%, balanced accuracy: 46.4%, *F*_1_-score: 48.3%, and agreement with human raters: κ=−0.05) classified almost all cases as abnormal, yielding a sensitivity of 84% (21/25 true positives) but 41 false positives, for a specificity of only 8.9%. The diagnostic metrics of all models in this task are presented in Table 3.

**Table 3.** Measures regarding identification of intraventricular conduction block^a^.

Measure	OpenAI ChatGPT-4	OpenAI ChatGPT-5	Google Gemini 2.5	Microsoft Copilot	Claude Sonnet-4	Claude Opus-4.1
Diagnostic metrics
Sensitivity, % (95% CI)	84 (65.3‐93.6)	60 (40.7‐76.6)	68 (48.4‐82.8)	68 (48.4‐82.8)	56 (37.1‐73.3)	36 (20.2‐55.5)
Specificity, % (95% CI)	8.9 (3.5‐20.7)	75.6 (61.3‐85.8)	57.8 (43.3‐71)	31.1 (19.5‐45.7)	46.7 (32.9‐60.9)	73.3 (59-84)
PPV^b^, % (95% CI)	33.9 (23.3‐46.3)	57.7 (38.9‐74.5)	47.2 (32-63)	35.4 (23.4‐49.6)	36.8 (23.4‐52.7)	42.9 (24.5‐63.5)
NPV^c^, % (95% CI)	50 (21.5‐78.5)	77.3 (63‐87.2)	76.5 (60‐87.6)	63.6 (43‐80.3)	65.6 (48.3‐79.6)	67.3 (53.4‐78.8)
Accuracy, % (95% CI)	35.7 (25.5‐47.4)	70 (58.5‐79.5)	61.4 (49.7‐72)	44.3 (33.2‐55.9)	50 (38.6‐61.4)	60 (48.3‐70.7)
Balanced accuracy, % (95% CI)	46.4 (37.6‐54.1)	67.8 (55.6‐79)	62.9 (50.1‐74.3)	49.6 (37.8‐61.2)	51.3 (39‐63.6)	54.7 (43.4‐66.5)
*F*_1_-score (95% CI)	48.3 (34.6‐61.1)	58.8 (40.9‐73.3)	55.7 (39.2‐69.6)	46.6 (31.4‐61)	44.4 (28.1‐59.4)	39.1 (19.4‐56.6)
Agreement measure
κ^d^	−0.05	0.35	0.23	−0.01	0.02	0.10

a Performance of generalist vision-enabled large language models in detecting intraventricular conduction block from 70 deidentified 12-lead electrocardiograms collected during routine clinical care in a cardiology ward at the University Medical Center Göttingen, Germany, with expert consensus as the reference standard. Model inference was conducted in July–August 2025. Prevalence of intraventricular conduction block was 25/70 (36%).

b PPV: positive predictive value.

c NPV: negative predictive value.

d Agreement with expert interpretation measured using Cohen κ.

For the 25 cases with intraventricular conduction block, we further assessed whether models could identify the correct type. ChatGPT-4 and ChatGPT-5 performed best, each correctly classifying 11 (44%) of 25 cases. ChatGPT-4’s result likely reflected its frequent labeling of intraventricular conduction block and frequent attempts at type assignment. No other model exceeded 7 correct classifications. Performance was particularly low for Claude Opus-4.1 (1/25, 4%) and Claude Sonnet-4 (2/25, 8%). Conditional accuracy (given that a block was correctly detected) was 60% for ChatGPT-4 and ChatGPT-5 but ≤30% for all other models. Agreement with human experts was highest for ChatGPT-5 (Cohen κ=0.37), followed by Gemini 2.5 (κ=0.10), while all other models demonstrated very poor agreements (κ<.1).

### QTc Interval Prolongation

Prolonged QTc was present in 22 (31.4%) ECGs. Model accuracy for detecting QTc prolongation varied significantly (Cochran Q, *P*=.002). ChatGPT-5 and ChatGPT-4 (accuracies: 71.4% and 67.1%, balanced accuracies: 58.2% and 61.3%, *F*_1_-scores: 33.3% and 46.5%, and agreement with human raters: κ=0.20 and 0.23, respectively) demonstrated the highest metrics, while Gemini 2.5 had the lowest metrics (accuracy: 48.6%, balanced accuracy: 40.3%, *F*_1_-score: 18.2%, and agreement with human raters: κ=−0.19). Most of the reported accuracy reflected correct classification of normal cases, with relatively high specificities but very low sensitivities, poor agreement with human raters, and classification performance as reflected by the detailed metrics shown in Table 4.

**Table 4.** Measures regarding identification of corrected QT interval (QTc) prolongation^a^.

Measure	OpenAI ChatGPT-4	OpenAI ChatGPT-5	Google Gemini 2.5	Microsoft Copilot	Claude Sonnet-4	Claude Opus-4.1
Diagnostic metric
Sensitivity, % (95% CI)	45.5 (26.9‐65.3)	22.7 (10.1‐43.4)	18.2 (7.3‐38.5)	0 (0‐14.9)	0 (0‐14.9)	9.1 (2.5‐27.8)
Specificity, % (95% CI)	77.1 (63.5‐86.7)	93.8 (83.2‐97.9)	62.5 (48.4‐74.8)	100 (92.6‐100)	97.9 (89.1‐99.6)	91.7 (80.4‐96.7)
PPV^b^, % (95% CI)	47.6 (28.3‐67.6)	62.5 (30.6‐86.3)	18.2 (7.3‐38.5)	NA^c^	0 (0‐79.3)	33.3 (9.7‐70)
NPV^d^, % (95% CI)	75.5 (61.9‐85.4)	72.6 (60.4‐82.1)	62.5 (48.4‐74.8)	68.6 (57‐78.2)	68.1 (56.4‐77.9)	68.8 (56.6‐78.8)
Accuracy, % (95% CI)	67.1 (55.5‐77)	71.4 (59.9‐80.7)	48.6 (37.2‐60)	68.6 (57‐78.2)	67.1 (55.5‐77)	65.7 (54‐75.8)
Balanced accuracy, % (95% CI)	61.3 (49.9‐73.2)	58.2 (49.3‐69)	40.3 (29.9‐51.5)	50 (50‐50)	49 (46.5‐50)	50.4 (43.9‐58.1)
*F*_1_-score (95% CI)	46.5 (27.3‐64)	33.3 (9.5‐54.5)	18.2 (4.9‐33.3)	NA	NA	14.3 (6.2‐33.3)
Agreement measure
κ^e^	0.23	0.20	−0.19	0	−0.03	0.01

a Performance of generalist vision-enabled large language models in detecting QTc prolongation from 70 deidentified 12-lead electrocardiograms collected during routine clinical care in a cardiology ward at the University Medical Center Göttingen, Germany, with expert consensus as the reference standard. Model inference was conducted in July–August 2025. Prevalence of QTc prolongation was 22/70 (31%).

b PPV: positive predictive value.

c NA: not applicable because no positives and/or no positive predictions.

d NPV: negative predictive value.

e Agreement with expert interpretation measured using Cohen κ.

### Premature Atrial and Ventricular Contractions

Only 3 ECGs contained PACs. None of the generalist models correctly detected these occurrences. Apparent accuracy for PAC detection was high (94%‐96%) in most models, but this was largely driven by the predominance of true negatives. All models except ChatGPT-5 answered “no” for every case (majority class classifier behavior), correctly classifying the 67 negatives but missing all 3 positives. ChatGPT-5 produced one false positive. Agreement with human experts was uniformly poor, with all models showing Cohen κ≤0. However, with only 3 PAC cases in the dataset, these estimates are unstable and do not permit reliable model-to-model comparison.

Ten ECGs contained at least one PVC. Sensitivity was again low. Only ChatGPT-5 detected any PVCs, correctly identifying 1 of 10 cases (sensitivity 10%) without false positives (specificity 100%). Gemini 2.5 produced one false positive, while most models (ChatGPT-4, Copilot, Claude Sonnet-4, and Claude Opus-4.1) answered “no” for all cases, yielding 100% specificity but 0% sensitivity. Cohen κ was≤0 for all models except ChatGPT-5 (κ=0.16). Calculated diagnostic metrics for PACs and PVCs are reported in Tables S8 and S9 in Multimedia Appendix 5.

### ST-Segment Deviation

Significant ST-segment deviations were present in 14 ECGs. Gemini 2.5 flagged ST changes in 16 cases, identifying 3 true positives but generating 13 false positives, resulting in 65.7% accuracy, 49.1% balanced accuracy, and an *F*_1_-score of 20%. In contrast, Copilot and Sonnet-4 were highly conservative, answering “no” in all cases (majority class classifier behavior) and yielding 80% accuracy. ChatGPT-4 detected 1 of 14 cases (sensitivity 7.1%) and Opus-4.1 had 0% sensitivity, although produced one false positive. ChatGPT-5 detected 2 of 14 cases (sensitivity 14.3%) with only one false positive, achieving the highest overall accuracy (81.4%) and balanced accuracy (56.2%) among generalist models. No model achieved sensitivity above 25%. Agreement with human experts was poor: all models showed κ≤0 (below chance level), except ChatGPT-5 (κ=0.18). All calculated diagnostic metrics for ST-segment deviations are provided in Table S10 in Multimedia Appendix 5.

### QRS Axis Deviation

Axis deviations were present in 12 cases. ChatGPT-5 demonstrated the highest sensitivity (66.7%, correctly identifying 8 of 12 abnormal axes) but the lowest specificity (75.9%, 14 false positives among 58 normal cases), resulting in an overall accuracy of 74.3% (balanced accuracy: 71.3%; *F*_1_-score=47.1%, and agreement with human experts: κ=0.32). In contrast, Claude Sonnet-4 and Claude Opus-4.1 each produced only one false positive, achieving 81.4% accuracy (balanced accuracy: 49.1% and agreement with human experts: κ=−0.03). The Cochran Q test indicated no significant differences among models (*P*=.097). The diagnostic metrics of all generalist models on this task are shown in Table 5.

**Table 5.** Measures regarding identification of QRS axis deviation^a^.

Measures	OpenAI ChatGPT-4	OpenAI ChatGPT-5	Google Gemini 2.5	Microsoft Copilot	Claude Sonnet-4	Claude Opus-4.1
Diagnostic metric
Sensitivity, % (95% CI)	25 (8.9‐53.2)	66.7 (39.1‐86.2)	41.7 (19.3‐68)	16.7 (4.7‐44.8)	0 (0‐24.2)	0 (0‐24.2)
Specificity, % (95% CI)	89.7 (79.2‐95.2)	75.9 (63.5‐85)	86.2 (75.1‐92.8)	89.7 (79.2‐95.2)	98.3 (90.9‐99.7)	98.3 (90.9‐99.7)
PPV^b^, % (95% CI)	33.3 (12.1‐64.6)	36.4 (19.7‐57)	38.5 (17.7‐64.5)	25 (7.1‐59.1)	0 (0‐79.3)	0 (0‐79.3)
NPV^c^, % (95% CI)	85.2 (74.3‐92)	91.7 (80.4‐96.7)	87.7 (76.8‐93.9)	83.9 (72.8‐91)	82.6 (72‐89.8)	82.6 (72‐89.8)
Accuracy, % (95% CI)	78.6 (67.6‐86.6)	74.3 (63‐83.1)	78.6 (67.6‐86.6)	77.1 (66‐85.4)	81.4 (70.8‐88.8)	81.4 (70.8‐88.8)
Balanced accuracy, % (95% CI)	57.3 (44.4‐70.5)	71.3 (55.5‐85.3)	63.9 (49‐79.2)	53.2 (43‐65.7)	49.1 (47.3‐50)	49.1 (47.2‐50)
*F*_1_-score (95% CI)	28.6 (9.5‐52.6)	47.1 (23.1‐66.7)	40 (14.7‐63.2)	20 (8.3‐43.9)	NA^d^	NA
Agreement measure
κ^e^	0.16	0.32	0.27	0.07	−0.03	−0.03

a Performance of generalist vision-enabled large language models in detecting QRS axis deviation from 70 deidentified 12-lead electrocardiograms collected during routine clinical care in a cardiology ward at the University Medical Center Göttingen, Germany, with expert consensus as the reference standard. Model inference was conducted in July to August 2025. Prevalence of QRS axis deviation was 12/70 (17%).

b PPV: positive predictive value.

c NPV: negative predictive value.

d NA: not applicable because no positives and/or no positive predictions.

e Agreement with expert interpretation measured using Cohen κ.

### Safety Analysis: False-Negative Errors in High-Priority ECG Abnormalities

To complement aggregate performance metrics, we analyzed false-negative classifications, defined as cases in which an abnormal ECG finding was labeled as normal, in high-priority abnormalities. For ST-segment deviation (n=14), generalist models missed between 11 and 14 cases, corresponding to false-negative rates of 79% to 100%. For AF (n=9), most generalist models missed all 9 cases (100% false-negative rate), with only 2 models detecting 1 of 9 cases (89% false-negative rate). For QT prolongation (n=22), false-negative counts ranged from 12 to 22 cases (55% to 100%) across models, indicating substantial underdetection. Although the number of positive cases was limited, these findings indicate that models frequently failed to identify abnormalities with potential immediate clinical consequences and that acceptable overall accuracy often masked a substantial burden of missed pathological findings.

### Performance of ECG-Specialized LLMs

To also explore the performance of ECG-specialized LLMs on our dataset, we tested 2 further models: PULSE-7B and ECG-Instruct-Llama-3.2-11B-Vision (ECG-Instruct-Llama-3.2). PULSE-7B demonstrated higher overall balanced accuracy (64.4% vs 56.5%) and *F*_1_-score (40.1% vs 32.9%) than ECG-Instruct-Llama-3.2 as detailed inTable 6. In paired analysis, discordant classifications numerically favored PULSE-7B (odds ratio [OR] 1.4, 95% CI 1.1‐1.8), although this asymmetry did not remain statistically significant after Bonferroni correction (adjusted *P*=.51). Among all generalist and specialized models, PULSE-7B achieved the highest overall balanced accuracy and *F*_1_-score, exceeding the best-performing general model, ChatGPT-5 (64.4% and 40.1% vs 61.8% and 38%, respectively). Paired McNemar testing between ChatGPT-5 and PULSE-7B demonstrated statistically significant asymmetry in discordant classifications (OR 2, 95% CI 1.45‐2.81; adjusted *P*=.001), indicating that ChatGPT-5 and PULSE-7B differed systematically at the case level. When comparing models with the highest balanced accuracy per diagnostic categories between the generalist and specialized groups, the generalist models, mostly ChatGPT-5, showed numerically higher balanced accuracy than the specialized models in the classification of first-degree AV Block, IV Block, IV Block Type, QT prolongation, ischemic ST-segment changes, and axis deviation. Head-to-head McNemar testing showed variable effect sizes (ORs) and mostly no statistical significance after correcting for multiple testing as shown in Table S11 in Multimedia Appendix 5.

**Table 6.** Overall measures of electrocardiogram (ECG)–specialized large language models^a^.

Measures	PULSE-7B	PULSE-7B (SP^b^)	ECG-Instruct-Llama-3.2	ECG-Instruct-Llama-3.2 (SP)
Diagnostic metric
Sensitivity, % (95% CI)	56.1 (47‐64.9)	60.6 (51.9‐68.7)	45.6 (37.1‐54.3)	42.1 (33.8‐50.8)
Specificity, % (95% CI)	72.7 (68.7‐76.3)	77.7 (73.9‐81.2)	67.3 (63.1‐71.3)	68.7 (64.5‐72.5)
PPV^c^, % (95% CI)	31.2 (25.3‐37.9)	40.7 (34‐47.9)	25.7 (20.4‐31.8)	25.1 (19.7‐31.4)
NPV^d^, % (95% CI)	88.2 (84.8‐91)	88.7 (85.4‐91.3)	83.3 (79.4‐86.6)	82.6 (78.7‐85.9)
Accuracy, % (95% CI)	69.7 (66‐73.1)	74.3 (70.7‐77.5)	63 (59.2‐66.7)	63.3 (59.5‐67)
Balanced accuracy, % (95% CI)	64.4 (59.6‐69.3)	69.2 (64.3‐73.8)	56.5 (51.4‐61.2)	55.4 (50.4‐60.2)
*F*_1_-score (95% CI)	40.1 (33.1‐46.9)	48.7 (41.7‐55.3)	32.9 (26.6‐39)	31.5 (24.8‐37.6)
Agreement measure
κ^e^	0.28	0.36	0.14	0.13

a Performance of image-based ECG-specialized vision-enabled large language models in interpreting 70 deidentified 12-lead ECGs collected during routine clinical care in a cardiology ward at the University Medical Center Göttingen, Germany, with expert consensus as the reference standard. Model inference was conducted in January 2026.

b SP: short-prompted.

c PPV: positive predictive value.

d NPV: negative predictive value.

e Agreement with expert interpretation measured using Cohen κ.

The use of an impression-style shortened prompt further increased the balanced accuracy and *F*_1_-score of PULSE-7B to 69.2% and 48.7%, respectively. Although discordant classifications numerically differed between prompt formats (OR 0.7, 95% CI 0.5‐0.9), this effect was not statistically significant after correction for multiple comparisons (adjusted *P*>.99). No prompt-related effect was observed for ECG-Instruct-Llama-3.2, where balanced accuracy and *F*_1_-score were similar using the standard and shortened prompts (56.5% vs 55.4% and 32.9% vs 31.5%, respectively; OR 1.0, 95% CI 0.7‐1.3; adjusted *P*>.99). When the short prompt was used for specialized models, they, mostly PULSE-7B, demonstrated numerically higher balanced accuracy than the generalist models in the classification of rhythm, first-degree AV Block, PACs, PVCs, and axis deviation as shown in Table S12 in Multimedia Appendix 5. However, this comparison remains limited because different prompts were used to test the models. Notably, the specialized models achieved consistently higher metrics regarding the detection of AF, with the short-prompted PULSE-7B reaching a balanced accuracy of 80.9% and an *F*_1_-score of 66.7%; however, this finding is still limited by the small case numbers in the tested dataset (9/70). All diagnostic metrics regarding the detection of AF are reported in Multimedia Appendix 7. When compared to a majority class classifier (ZeroR) across all categories, both standard-prompted ECG-specialized models had accuracy below or equal to ZeroR accuracy. In McNemar testing, no statistically significant asymmetry in discordant classifications was observed in all categories except for the detection of first-degree AV Block with PULSE-7B (OR 7.5, 95% CI 1.7‐32.8, adjusted *P*=.04) and ischemic ST-segment changes in ECG-Instruct-Llama-3.2 (OR 3.5, 95% CI 1.9‐6.6, adjusted *P*<.001). The only model that had favorable and statistically significant asymmetry in discordant classifications in comparison with ZeroR was the short-prompted PULSE-7B in PVC detection (OR 0.1, 95% CI 0‐1, unadjusted *P*=.046), correlating with the highest balanced accuracy and *F*_1_-score among all generalist and specialized models for this task (89.2% and 84.2%, respectively). However, this superiority did not survive correction for multiple testing (adjusted *P=*.455) as shown in Multimedia Appendix 6.

## Discussion

### Summary of Findings

To the best of our knowledge, this is the first broad evaluation of the latest publicly available VE-LLMs on image-based ECG interpretation. We found substantial variability in performance and response characteristics across the 6 generalist models tested. Overall balanced accuracy ranged from 50.1% to 61.8%, with ChatGPT-5 achieving the highest overall balanced accuracy across the 9 ECG question categories. All generalist models correctly identified the majority class (normal SR or normal findings) in most cases but performed poorly on less common abnormalities. Sensitivity was particularly low for AF, conduction blocks, and ectopic beats, despite high specificity driven by a tendency toward normal classifications. This resulted in only poor or, at best, fair agreement with human expert answers on most tasks. The image-based ECG-specialized model, PULSE-7B, demonstrated slightly higher overall balanced accuracy than generalist models (64.4%). PULSE-7B showed numerically higher task-specific performance metrics for rhythm classification, identification of AF, and ectopic beats. However, this pattern was not consistent across other diagnostic categories (Summary Figure in Multimedia Appendix 8). These findings should be interpreted as descriptive and hypothesis generating, given the small number of positive cases in multiple categories.

### Performance of Publicly Accessible Vision-Enabled LLMs in Image-Based ECG Analysis

Our findings align with and extend early investigations of multimodal LLMs for ECG analysis. A recently published study evaluated ChatGPT-4 on 6 ECG categories. With the zero-shot approach and no textual guidance, ChatGPT-4 achieved an accuracy of 53% in detecting abnormal ECGs, resulting mainly from a perfect sensitivity (100%), at the cost of a very poor specificity (7%). Providing textual guidance could boost the accuracy up to 63%. ChatGPT-4 also showed poor performance identifying specific pathologies even with textual guidance with an accuracy ranging from 28% to 41% [13]. This difficulty in multidiagnosis interpretation mirrors our results, where no model reliably recognized all the diverse ECG abnormalities. Another study by Zeljkovic et al [14] yielded comparable performance metrics; however, it demonstrated that clinical context can significantly improve accuracy of ChatGPT-4 interpretation of ECGs (from 19% to 45%). However, contextual information alone is unlikely to overcome intrinsic limitations of current vision encoders, particularly for tasks requiring precise measurements. Beyond ChatGPT-4, only very few studies investigated the performance of other generalist VE-LLMs in ECG interpretation; 2 comparative studies have shown that ChatGPT-4 outperformed Gemini, with both performing substantially below human experts [9,15]. Our study is the first to investigate the state-of-the-art OpenAI model, ChatGPT-5, and to broadly compare the performance of generalist and 2 image-based ECG-specialized LLMs, PULSE-7B and ECG-Instruct-Llama-3.2. In their original reports, both specialist models demonstrated improved performance compared with general multimodal LLMs on ECG image benchmarks [11,12]. In our external dataset, the overall performance metrics of PULSE-7B were slightly higher than generalist models; however, this superiority was rather modest and not consistent across all diagnostic categories. PULSE-7B achieved an overall balanced accuracy of 64.4%, which could be boosted to 69.2% with the impression-style concise prompt, whereas ECG-Instruct-Llama-3.2 reached approximately 56% overall balanced accuracy with limited improvement with prompt shortening. Notably, ECG-specialized models achieved the highest performance metrics in rhythm classification. Besides, the short-prompted PULSE-7B reached high balanced accuracy (up to 89.2%) and good agreement with expert annotations (up to 0.82) regarding the detection of ectopic beats. This may reflect its instruction-tuning on large ECG image corpora emphasizing visual pattern recognition of rhythm irregularity and beat morphology, rather than interval measurements that depend more strongly on precise signal decoding and fine-grained temporal resolution, which may be less robust in image-based inference pipelines.

In contrast to image-based LLMs, several deep learning CNNs trained specifically on ECG waveforms have already achieved expert-level performance [16] and, in some cases, identified hidden patterns that are not apparent to human readers [8]. For example, specialized ECG CNN models can predict AF in patients with SR [6,7]. In our dataset and study settings, generalist VE-LLMs failed to reliably detect the presence of AF based on ECG images, with a task-specific advantage for specialized models, which reached a markedly higher balanced accuracy and *F*_1_-scores for AF when compared with the generalist models. A recent head-to-head comparison found that a specialized ECG diagnostic model (“ECG Buddy”) significantly outperformed ChatGPT-4 in detecting acute myocardial infarction on ECGs [15]. We did not observe a similar trend for ischemic ST-segment deviation in our study, where both generalist and specialized LLMs performed poorly and the highest balanced accuracy (56.2%) was achieved by ChatGPT-5. Although ECG-Instruct-Llama-3.2 scored the highest sensitivity (92.9%) in this task, this was at the cost of a very low specificity (17.9%), resulting from its tendency to label most presented ECGs as suspicious for ischemia. Conversely, many VE-LLMs achieved seemingly high accuracy in some subcategories, largely driven by class imbalance and correct classification of normal findings, as the majority class (ZeroR) baseline achieved higher overall accuracy than most models, suggesting potentially limited incremental value beyond predicting the most frequent class. However, this apparent advantage reflects class imbalance rather than meaningful diagnostic performance: because ZeroR always predicts the majority class, balanced accuracy remains at the chance level of 50%, and in categories where the majority class corresponds to normal findings the *F*_1_-score collapses to 0, indicating no clinical utility for abnormality detection. Agreement of generalist models with human experts was consistently poor: κ values were often near or below zero, reflecting little true concordance beyond chance. Taken together, these findings indicate that current general-purpose VE-LLMs remain markedly inferior to human experts for detailed ECG interpretation.

### Challenges of Current Vision-Enabled LLMs in ECG Interpretation

Several factors may explain why generalist cutting-edge LLMs underperformed in image-based ECG interpretation despite their strong language abilities. A key issue is training data. These models were trained on vast datasets that likely contained relatively few annotated ECG images, as such data are not abundant in the public domain compared with natural images or text. Consequently, their vision encoders may not have developed the specialized feature detectors required for precise waveform analysis, limiting their ability to capture the fine-grained features essential for ECG interpretation. Zhu et al [10] reported that ChatGPT-4 could answer roughly two-thirds of multiple-choice ECG questions correctly, but it struggled disproportionately with questions requiring precise waveform measurements (eg, identifying a prolonged PR interval). We similarly found that first-degree AV block (prolonged PR) and QT prolongation were seldom detected by the tested models, possibly because ECG images, even when exported at high resolution, are internally downsampled or tokenized by vision language models, reducing fine waveform detail. As a result, precise measurement of interval durations (eg, PR or QT) may become technically challenging, particularly when interpretation depends on millimeter-scale grid resolution.

Although hallucinations were not systematically captured or quantified in this study, they remain a major safety issue for medical applications of LLMs. Pesapane et al [17] documented multiple examples of ChatGPT-4 inventing abnormalities on normal mammograms. A recently published study that evaluated the performance of ChatGPT-4 and Gemini 1.5 in ECG interpretation with a focus on different types of hallucination revealed that even when LLMs answered correctly, hallucinations were still common [18]. Thus, measuring LLM performance solely by the percentage of correct responses on multiple-choice tests may overestimate true interpretive ability.

The modest performance of ECG-specialized VE-LLMs in our evaluation compared with benchmark reports likely reflects several factors. Our study used real-world cardiology-ward ECG exports, whereas training and benchmark datasets included synthetically rendered ECG images derived from signal data and different cohorts. In addition, unlike prior reports, our zero-shot prompting approach required strict categorical decisions based on predefined measurement thresholds. Moreover, these comparisons are not strictly head-to-head, as prior studies used different datasets, label definitions, and evaluation protocols than our fixed 9-question rubric applied to real-world ECG image exports. Finally, the modest sample size, class imbalance, and low prevalence of certain abnormalities likely affected performance metrics.

### Study Limitations

This study has several limitations. First, the dataset was small (70 ECGs) and lacked rare but important findings such as ventricular tachycardia or advanced AV block. The study was not powered to detect predefined differences between models. A post-hoc minimum detectable difference analysis indicated that only absolute differences in overall accuracy ≥22% could be detected with adequate statistical power. Smaller effect sizes may therefore not have been detectable, and findings should be interpreted as descriptive and hypothesis generating. In addition, the dataset was derived from a dedicated heart rhythm ward, where the pretest probability of arrhythmias and conduction abnormalities is higher than in general screening or emergency department populations. This case mix may have influenced predictive values and limits the generalizability of our findings to broader clinical settings and patient populations. Second, because all models were evaluated using a standardized zero-shot prompt, the reported results likely reflect baseline performance rather than maximum achievable capability. Advanced prompting strategies (eg, few-shot or chain-of-thought prompting) might have improved accuracy, as shown in previous research [13,14]. Third, the ECG-Instruct-Llama-3.2-11B-Vision model required manual extraction and mapping of diagnostic findings from narrative outputs; although done with a conservative approach, some degree of interpretation bias and misclassification cannot be fully excluded. In addition, output generation for the ECG-specialized models was limited to 512 new tokens (Table S2 in Multimedia Appendix 1), which may have truncated longer responses and reduced sensitivity if abnormalities were not included in the generated output. Fourth, some diagnostic categories were imbalanced in prevalence, which may have inflated accuracy metrics. Fifth, our evaluation was limited to English prompts; performance may differ in other languages or formats. Sixth, hallucinations were not systematically captured or quantified in this study; therefore, qualitative assessment of such errors was beyond the scope of the present analysis. Seventh, reference answers were defined by 2 cardiologists, which is reliable, although not infallible. We focused on well-quantifiable gross abnormalities, where careful measurement and consensus reading make the likelihood of missed findings very low. In addition, expert readers performed manual interval measurements using full-resolution ECG images, whereas vision-enabled multimodal models internally downsample and tokenize image inputs, creating an inherent asymmetry that may disadvantage models in interval-dependent tasks. Eighth, we did not include a conventional signal-based CNN benchmark; therefore, direct comparison with established waveform-based AI systems is not possible. Ninth, response time measurements were obtained via web interfaces and may therefore have been influenced by factors other than intrinsic model inference speed, such as server load, account tier, and time of day. In addition, the generalist web-based models were evaluated from July to August 2025, whereas the ECG-specialized models were tested in January 2026. As publicly deployed multimodal systems may undergo unannounced backend updates over time, comparisons across these evaluation windows should be interpreted cautiously. Finally, although we included the most advanced VE-LLMs at the time of study, these systems evolve rapidly, so our results may not generalize to future updates.

### Clinical Translation and Future Research Directions

Recent developments underscore both the promise and the challenge of combining LLM structures with dedicated ECG encoding and training. For example, Yang et al [19] introduced ECG-LM as the first cross-modal LLM aligned with a dedicated ECG encoder, which achieved remarkable zero-shot results in ECG diagnostics and question-answering. In a very recent preprint, Xia et al [20] have applied a different approach and proposed ECG-aBcDe, a framework that encodes ECGs into a universal representation consumable by LLMs. In this early evaluation, ECG-aBcDe demonstrated substantially improved zero-shot performance in ECG question-answering across datasets compared with prior approaches. Notably, these solutions are still largely based on ECG signal data. Effective translation of LLM technologies into routine clinical workflows requires a feasible and reliable image-based interpretation, a capability that has not yet been established. Beyond the specific model comparisons, the standardized evaluation pipeline presented here—including structured prompting, predefined diagnostic tasks, and detailed reporting of inference settings—may serve as a reproducible framework for benchmarking future vision-enabled LLMs as the field evolves.

### Conclusions

Our work provides a timely reality check on the capabilities of generalist multimodal LLMs in a core cardiology task, ECG interpretation. While their language abilities are remarkable, the performance of generalist LLMs in ECG interpretation is inconsistent across models and diagnostic categories and remains insufficient for safe clinical use. ChatGPT-5 achieved the highest overall accuracy among publicly accessible VE-LLMs, yet it too fell short of expert standards. The ECG-specialized model, PULSE-7B, demonstrated a slightly better overall performance and may offer advantages in selected tasks, but the performance is still inconsistent across the full range of diagnostic categories. Although promising, current evidence indicates that VE-LLMs require further optimization and validation before routine clinical integration.

## Supplementary material

10.2196/86692 Multimedia Appendix 1 Inference environment and settings used for the evaluated models.

10.2196/86692 Multimedia Appendix 2 Example interaction with ChatGPT-5 demonstrating the model query and response process (screen recording).

10.2196/86692 Multimedia Appendix 3 R scripts used for data processing, statistical analyses, and generation of study figures

10.2196/86692 Multimedia Appendix 4 Dataset matrix.

10.2196/86692 Multimedia Appendix 5 Extended results including additional tables and figures detailing model performance and diagnostic metrics across evaluation tasks.

10.2196/86692 Multimedia Appendix 6 Comparison of model predictions with the majority class classifier (ZeroR) across all diagnostic categories.

10.2196/86692 Multimedia Appendix 7 Diagnostic performance metrics for atrial fibrillation detection across the evaluated models.

10.2196/86692 Multimedia Appendix 8 Performance of 6 publicly accessible generalist and 2 ECG-specialized vision-enabled large language models (VE-LLMs) in image-based ECG interpretation. Seventy ECG tracings from cardiology ward patients were submitted to VE-LLMs using a standardized detailed prompt, in addition to a short prompt (SP) in case of specialized VE-LLMs. Models’ outputs were compared against expert consensus across 9 diagnostic categories. The Results section (center) contains 2 heatmaps: the balanced accuracy heatmap (left) showing the balanced accuracies, and the Cohen κ heatmap (right) showing agreement with expert interpretation beyond chance level per model and diagnostic category.

## Abbreviations

AF: atrial fibrillation
AI: artificial intelligence
AV: atrioventricular
CNN: convolutional neural network
ECG: electrocardiogram
IVCD: intraventricular conduction delay
LLM: large language model
NPV: negative predictive value
OR: odds ratio
PAC: premature atrial contraction
PPV: positive predictive value
PVC: premature ventricular contraction
QTc: corrected QT interval
SR: sinus rhythm
USMLE: United States Medical Licensing Examination
VE-LLM: vision-enabled large language model
