# Supplementary material for: Performance of Vision-Enabled Large Language Models in Image-Based Electrocardiogram Interpretation: Exploratory Evaluation
Source: J Med Internet Res. 2026 Jun 3;28:e86692. doi: 10.2196/86692 (PMC13234008; doi:10.2196/86692)
Supplement: Multimedia Appendix 1 [file jmir-v28-e86692-s001.docx]

**Multimedia Appendix 1
(Methods)**

**Article:**

**Performance of Vision-Enabled Large Language Models in Image-based ECG Interpretation: Exploratory Evaluation**

Nibras Soubh, Eva Rasenack, Helge Haarmann, Felix Wiedmann, Markus Zabel, Constanze Schmidt, Rayan Suliman*, Leonard Bergau*

* Equal contribution

**Summary:**Performance of vision-enabled large language models in image-based interpretation of 70 de-identified 12-lead ECGs collected during routine clinical care in a cardiology ward at the University Medical Center Göttingen, Germany, with expert consensus as the reference standard. Model inference was conducted in July–August 2025 (generalist models) and January 2026 (specialized models).

**Table of Contents**

**Table S1:** Inference Dates and Settings of The Generalist LLMs …………………………. ……… 2

**Table S2:** Environment and Inference Settings of ECG Specialized Models ………….. ……… 3

**Table S1:** Inference Dates and Settings of Generalist LLMs **^†^**

| **Model** | **Provider** | **Inference Dates** | **Inference Platform** (Subscription Level) | **Settings*^a^*** |
| --- | --- | --- | --- | --- |
| **ChatGPT-4** | OpenAI | 29.07.2025 - 08.08.2025 | chatgpt.com (Plus) | Default “4o model” |
| **ChatGPT-5** | OpenAI | 10.08.2025 - 15.08.2025 | chatgpt.com (Plus) | Default |
| **Gemini-2.5** | Google | 30.07.2025 - 12.08.2025 | gemini.google.com (Pro) | Default |
| **Copilot** | Microsoft | 29.07.2025 - 17.08.2025 | copilot.microsoft.com (Free) | “Think Deeper” |
| **Claude Sonnet-4** | Anthropic | 29.07.2025 - 12.08.2025 | claude.ai (Pro) | Default |
| **Claude Opus-4.1** | Anthropic | 29.07.2025 - 12.08.2025 | claude.ai (Pro) | Default |
| **Grok-4** | xAI | 29.07.2025 - 15.08.2025 | perplexity.ai (Pro) | **Excluded from analysis *** |

LLM, large language model

**^†^** Generalist models were evaluated in July-August 2025, whereas ECG-specialized models were evaluated in January 2026. Owing to the rapid update cycles of multimodal systems and possible unannounced backend changes, comparisons across these evaluation windows should be interpreted cautiously.

***^a^*** All interactions were performed using a standardized local client environment and browser (Google Chrome). The hardware configuration consisted of a Lenovo Yoga Pro 9 (16IMH9) equipped with an Intel Core Ultra 9 185H processor (2.30 GHz) and 32 GB of RAM, running Windows 11 Home (Version 25H2). Geographic location: Göttingen, Lower Saxony, Germany.

***** Data initially generated using Grok-4 (xAI) via the Perplexity Pro platform in July and August 2025 were excluded from the final analysis. This decision was made following the retrospective disclosure of a 'silent fallback bug' by the platform provider. Due to this technical issue, queries intended for Grok-4 were intermittently routed to GPT-4 without user notification, confounding the model-specific results.

**Table S2:** Environment and Inference Settings of ECG-Specialized Models **^†^**

|  | **PULSE-7B** | **ECG-Instruct- Llama-3.2-11B-Vision:** |
| --- | --- | --- |
| **Computing environment** | Google Colab Pro+ | |
| **GPU** | NVIDIA A100 Tensor Core GPU (40 GB VRAM) | |
| **Numerical Precision** | BFloat16 | FP16 |
| **Quantization** | None | |
| **Software stack** | Python 3.10, PyTorch 2.x, LLaVA framework | Python 3.10, PyTorch 2.x, Hugging Face Transformers |
| **Image format** | JPEG ECG images, converted to RGB (PIL), single-image inference | |
| **Decoding strategy** | Greedy decoding | |
| **Inference Settings** |  | |
| **- Temperature** | 0.0 ***^a^*** | |
| **- Top-p** | 1.0 ***^a^*** | |
| **- Sampling** | Disabled ***^a^*** | |
| **- Max New Tokens** | 512 ***^a^*** | |
| **- Determinism** | Not enforced | |

GPU, Graphics processing unit.

**^†^** Generalist models were evaluated in July-August 2025, whereas ECG-specialized models were evaluated in January 2026. Owing to the rapid update cycles of multimodal systems and possible unannounced backend changes, comparisons across these evaluation windows should be interpreted cautiously.

***^a^*** Parameters also explicitly specified in the inference execution command.
