# Supplementary material for: Performance of Vision-Enabled Large Language Models in Image-Based Electrocardiogram Interpretation: Exploratory Evaluation
Source: J Med Internet Res. 2026 Jun 3;28:e86692. doi: 10.2196/86692 (PMC13234008; doi:10.2196/86692)
Supplement: Multimedia Appendix 5 [file jmir-v28-e86692-s005.docx]

**Multimedia Appendix 5
(Extended Results)**

**Article:**

**Performance of Vision-Enabled Large Language Models in Image-based ECG Interpretation: Exploratory Evaluation**

Nibras Soubh, Eva Rasenack, Helge Haarmann, Felix Wiedmann, Markus Zabel, Constanze Schmidt, Rayan Suliman*, Leonard Bergau*

* Equal contribution

**Summary:**Performance of vision-enabled large language models in image-based interpretation of 70 de-identified 12-lead ECGs collected during routine clinical care in a cardiology ward at the University Medical Center Göttingen, Germany, with expert consensus as the reference standard. Model inference was conducted in July–August 2025 (generalist models) and January 2026 (specialized models).

**Table of Contents**

**Table S3:** Inter-Rater Metrics ………………………………………………………………………………… 2

**Table S4:** Overall Performance Metrics of Generalist Models …………………………………….. 3

**Table S5:** Comparison of Overall Accuracy with Majority-Class Classifier (ZeroR).…………. 4

**Figure S1:** Accuracy Heatmap ………………………………………………………………………………………………… 5

**Table S6:** Overall Accuracy Across Difficulty Spectrum……………………………………………………………. 6
**Table S7:** Overall Balanced Accuracies and F1 Scores Across Difficulty Spectrum …………………… 7
**Table S8:** Diagnostic Metrics Regarding Identification of PACs ……………………………………………….. 8

**Table S9:** Diagnostic Metrics Regarding Identification of PVCs ……………………………………………….. 9

**Table S10:** Diagnostic Metrics Regarding Identification of significant ST-Segment deviation …… 10

**Table S11:** Comparison of Best Performing Generalist and Specialized LLMs Based

on Balanced Accuracy Using an Identical Long Prompt……………………………………………………………. 11

**Table S12:** Comparison of Best Performing Generalist and short-prompted Specialized

LLMs Based on Balanced Accuracy ………………………………………………………………………………………….. 12

**Table S3:** Inter-Rater Metrics

|  | **Agreement (%)** | **Cohen’s Kappa** | **Lower CI** | **Upper CI** |
| --- | --- | --- | --- | --- |
| **Rhythm** | 100 | 1 | 1 | 1 |
| **First degree AV Block** | 97.1 | 0.87 | 0.70 | 1 |
| **Intraventricular Block** | 97.1 | 0.94 | 0.85 | 1 |
| **IV-Block Type** | 95.7 | 0.92 | 0.82 | 1 |
| **QT-Prolongation** | 95.7 | 0.90 | 0.80 | 1 |
| **PACs** | 98.6 | 0.85 | 0.56 | 1 |
| **PVCs** | 100 | 1 | 1 | 1 |
| **Ischemic ST-Changes** | 94.2 | 0.80 | 0.61 | 0.99 |
| **Axis Deviation** | 97.1 | 0.91 | 0.78 | 1 |
| **All Categories** | **97.3** | 0.94 | 0.92 | 1 |

CI, confidence interval; AV, atrioventricular; IV, intraventricular; PACs, premature atrial contractions; PVCs, premature ventricular contractions

**Table S4:** Overall Performance Metrics of Generalist Models

|  | **OpenAI  ChatGPT-4** | **OpenAI  ChatGPT-5** | **Google  Gemini-2.5** | **Microsoft  Copilot** | **Claude  Sonnet-4** | **Claude  Opus-4.1** |
| --- | --- | --- | --- | --- | --- | --- |
| **Sensitivity** (%) (95% CI) | **42** (33.5-51) | 35.3  (27.3-44.2) | 29.4  (22-38.1) | 21  (14.7-29.2) | 14.9  (9.6-22.3) | 9.6  (5.6-16) |
| **Specificity** (%) (95% CI) | 75.9  (72-79.4) | **88.3**  (85.2-90.8) | 80.2  (76.6-83.5) | 83.6  (80.1-86.5) | 87.6  (84.5-90.2) | 90.7  (87.8-92.9) |
| **PPV** (%) (95% CI) | 28.9  (22.7-36.1) | **41.2**  (32.1-50.9) | 25.7  (19.1-33.7) | 22.9  (16-31.7) | 22.2  (14.5-32.4) | 20.3  (12-32.3) |
| **NPV** (%) (95% CI) | 84.9  (81.3-87.9) | **85.4**  (82.2-88.2) | 83  (79.4-86.1) | 82  (78.4-85) | 81.2  (77.8-84.3) | 80.2  (76.7-83.3) |
| **Accuracy** (%) (95% CI) | 69.5  (65.8-73) | **78.3**  (74.9-81.3) | 70.6  (67-74.1) | 71.7  (68.1-75.1) | 73.7  (70.1-76.9) | 74.6  (71.1-77.8) |
| **Balanced Accuracy** (%) (95% CI) | 59  (54.4-63.6) | **61.8**  (57.5-66.2) | 54.8  (50.6-59.4) | 52.3  (48.3-56.5) | 51.2  (48.1-55) | 50.1  (47.3-53.2) |
| **F1 score** (95% CI) | 34.2  (27.4-41) | **38**  (29.8-45.6) | 27.5  (20.3-34.6) | 21.9  (14.6-29) | 17.8  (11-25.1) | 13  (6.8-19.9) |
| *κ ^a^*= | 0.21 | **0.31** | 0.16 | 0.11 | 0.09 | 0.05 |

CI, confidence interval; PPV, positive predictive value; NPV, negative predictive value.

a κ: agreement with human experts using Cohen’s kappa test.

**Table S5:** Comparison of All Models with Majority-Class Rating (ZeroR)

|  | **Δ Accuracy (%)** | **Odds Ratio (95% CI)** | ***P**** |
| --- | --- | --- | --- |
| **Generalist LLMs** |  |  |  |
| ChatGPT-4 | -9.5 | 2.3 (1.6-3.2) | **<.001** |
| ChatGPT-5 | -1 | 1 (0.6-1.5) | 1 |
| Gemini-2.5 | -8.3 | 2.5 (1.7-3.7) | **<.001** |
| Copilot | -7.3 | 2.9 (1.8-4.6) | **<.001** |
| Claude Sonnet-4 | -5.4 | 3.1 (1.8-5.3) | **<.001** |
| Claude Opus-4.1 | -4.3 | 3.4 (1.8-6.4) | **.002** |
| **Specialized LLMs** |  |  |  |
| PULSE-7B | -9.3 | 2 (1.4-2.7) | **<.001** |
| PULSE-7B (SP) | -4.5 | 1.5 (1.1-2) | .106 |
| ECG-Instruct-Llama-3.2 | -16.1 | 2.6 (1.9-3.6) | **<.001** |
| ECG-Instruct-Llama-3.2 (SP) | -15.9 | 2.9 (2.1-4.1) | **<.001** |

LLMs, large language models; CI, confidence interval; SP, short-prompted
* McNemar test, adjusted for multiple testing with Bonferroni correction

**Figure S1:** Accuracy Heatmap


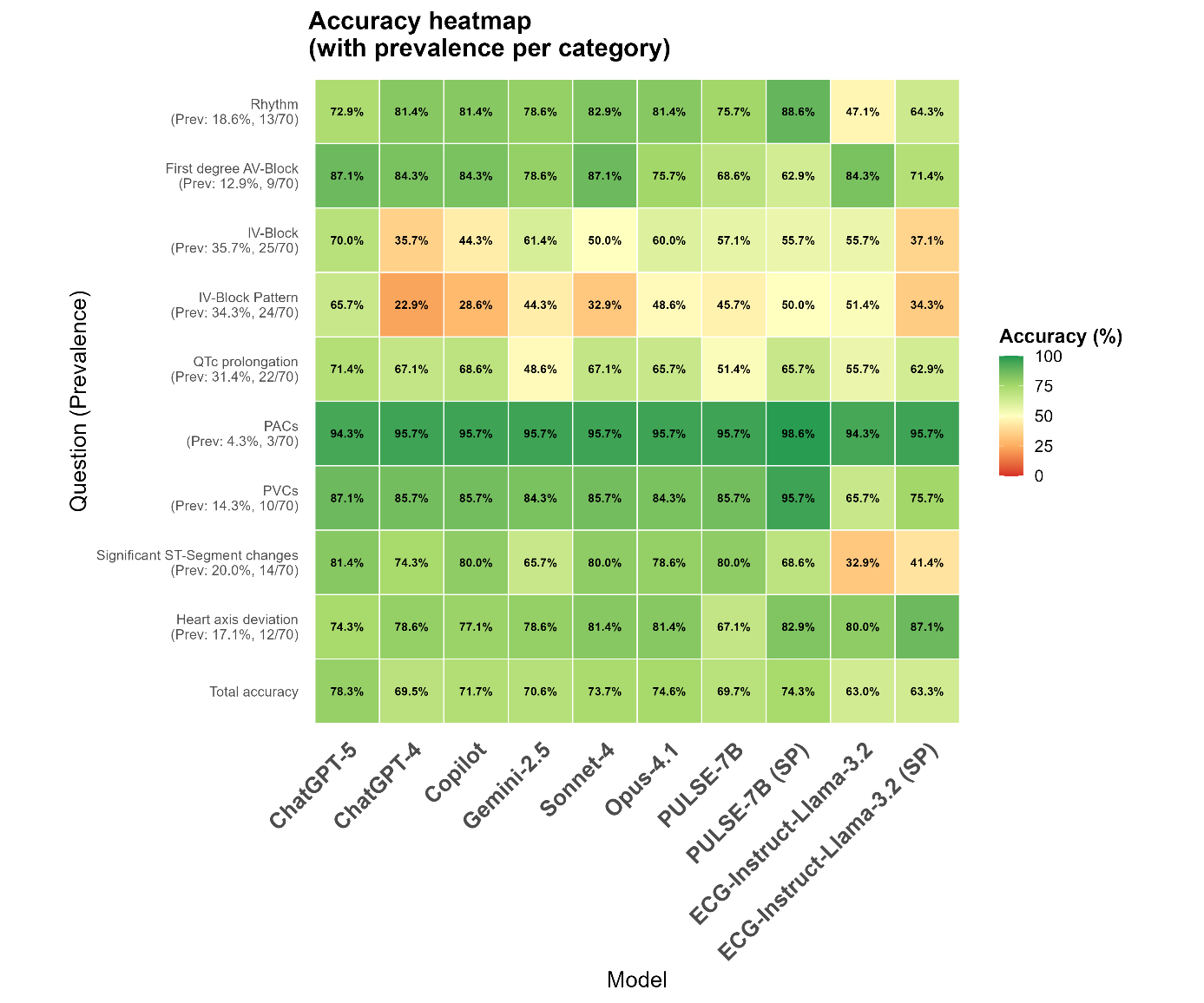


Accuracy heatmap comparing performance of all tested large language models across different interpretation tasks. Accuracy values are expressed as percentages and represented using a color scale (green = higher accuracy, orange/red = lower accuracy).

Prev, prevalence; AV, atrioventricular; IV, intraventricular; QTc, corrected QT interval; PACs, premature atrial contractions; PVCs, premature ventricular contractions; SP, short-prompted.

**Table S6:** Overall Accuracy Across Difficulty Spectrum

| **Accuracy per  Difficulty Level** | **OpenAI ChatGPT-4** | **OpenAI  ChatGPT-5** | **Google  Gemini- 2.5** | **Microsoft Copilot** | **Claude  Sonnet-4** | **Claude  Opus-4.1** |
| --- | --- | --- | --- | --- | --- | --- |
| **Level-1** (%) (95% CI) | 65.2 (58.6-71.4) | **81.9** (76.1-86.5) | 73.8 (67.5-79.3) | 75.2 (69-80.6) | 73.8 (67.5-79.3) | 79.5 (73.6-84.4) |
| **Level-2** (%) (95% CI) | 65.5 (60-70.6) | 68.4 (63-73.3) | 64.2 (58.7-69.3) | 64.9 (59.4-69.9) | 68.4 (63-73.3) | **66.5** (61-71.5) |
| **Level-3** (%) (95% CI) | 54.4 (47.1-61.5) | **60.6** (53.3-67.4) | 50.6 (43.3-57.8) | 51.7 (44.4-58.9) | 53.9 (46.6-61) | 53.9 (46.6-61) |
| ***P*=*** | **.043** | **<.001** | **<.001** | **<.001** | **<.001** | **<.001** |

CI, confidence interval
*: Per-model analyses using two-sided Pearson χ² on the 3×2 contingency tables (correct/incorrect) stratified according to difficulty

**Table S7:** Overall Balanced Accuracies and F1 Scores Across Difficulty Spectrum

| **Metrics per  Difficulty Level** | **OpenAI ChatGPT-4** | **OpenAI  ChatGPT-5** | **Google  Gemini-2.5** | **Microsoft Copilot** | **Claude  Sonnet-4** | **Claude  Opus-4.1** |
| --- | --- | --- | --- | --- | --- | --- |
| **Level-1** |  |  |  |  |  |  |
| Balanced Accuracy (%)  (95% CI) | 56.7 (43.1-71) | **70.2** **(55.8-84.7)** | 61.8 (48.1-76.6) | 61.6 (48.8-76.8) | 47.6 (42-56.4) | 51 (46.2-60) |
| F1 score (%)  (95% CI) | 16.1 (3.9-28.6) | **41.4 (16-62.9)** | 22.7  (5.9-38.8) | 24.4  (6.2-41.9) | 5.6  (0-17.4) | 8.3  (0-25) |
| **Level-2** |  |  |  |  |  |  |
| Balanced Accuracy (%)  (95% CI) | **61.6** (54.3-68.6) | 59.5 (53.1-66.2) | 56.8 (50-63.9) | 51.7 (46.3-57.8) | 55.6 (49.9-61.9) | 52.2 (47.1-57.6) |
| F1 score (%)  (95% CI) | **38.7**  **(27.2-49.1)** | 34.9  (22.6-46.6) | 31  (20.6-41.7) | 20.4  (9-31.2) | 26.4  (13.7-38.4) | 19.8  (8.8-30.1) |
| **Level-3** (%) |  |  |  |  |  |  |
| Balanced Accuracy (%)  (95% CI) | 55.4 (47.7-63.7) | **58.6** (50.8-66.7) | 47.3 (40-54.7) | 47.3 (40.7-54.4) | 47.5 (42.8-52.7) | 46.6 (42.5-50.4) |
| F1 score (%)  (95% CI) | 39.6 (28.3-51.6) | **40.5** (27-53.1) | 25.3 (13.3-36) | 22.5 (11-34.3) | 13.3 (3-24.4) | 5.8 (0-14.3) |

CI, confidence interval

**Table S8:** Diagnostic Metrics Regarding Identification of PACs

| **Metric** | **OpenAI ChatGPT-4** | **OpenAI  ChatGPT-5** | **Google  Gemini-2.5** | **Microsoft Copilot** | **Claude  Sonnet-4** | **Claude  Opus-4.1** |
| --- | --- | --- | --- | --- | --- | --- |
| **Sensitivity** (%) (95% CI) | 0  (0-56.1) | 0  (0-56.1) | 0  (0-56.1) | 0  (0-56.1) | 0  (0-56.1) | 0  (0-56.1) |
| **Specificity** (%) (95% CI) | 100  (94.6-100) | 98.5  (92-99.7) | 100  (94.6-100) | 100  (94.6-100) | 100  (94.6-100) | 100  (94.6-100) |
| **PPV** (%) (95% CI) | NA | 0  (0-79.3) | NA | NA | NA | NA |
| **NPV** (%) (95% CI) | 95.7  (88.1-98.5) | 95.7  (88-98.5) | 95.7  (88.1-98.5) | 95.7  (88.1-98.5) | 95.7  (88.1-98.5) | 95.7  (88.1-98.5) |
| **Accuracy** (%) (95% CI) | 95.7  (88.1-98.5) | 94.3  (86.2-97.8) | 95.7  (88.1-98.5) | 95.7  (88.1-98.5) | 95.7  (88.1-98.5) | 95.7  (88.1-98.5) |
| **Balanced Accuracy** (%) (95% CI)^*^ | 50  (50-50) | 49.3  (47.7-50) | 50  (50-50) | 50  (50-50) | 50  (50-50) | 50  (50-50) |
| **F1 score** (%) (95% CI) | NA | NA | NA | NA | NA | NA |

*Prevalence of PACs: 3/70* (4.3%)

CI, confidence interval; PPV, positive predictive value; NPV, negative predictive value.
NA: not applicable because no positives and/or no positive predictions.

^*^ In categories without true positive predictions, bootstrap confidence intervals for balanced accuracy may collapse to zero width because resampling cannot introduce variability into sensitivity estimates.

**Table S9:** Diagnostic Metrics Regarding Identification of PVCs

| **Metric** | **OpenAI ChatGPT-4** | **OpenAI  ChatGPT-5** | **Google  Gemini-2.5** | **Microsoft Copilot** | **Claude  Sonnet-4** | **Claude  Opus-4.1** |
| --- | --- | --- | --- | --- | --- | --- |
| **Sensitivity** (%) (95% CI) | 0  (0-27.8) | **10**  (1.8-40.4) | 0  (0-27.8) | 0  (0-27.8) | 0  (0-27.8) | 0  (0-27.8) |
| **Specificity** (%) (95% CI) | **100**  (94-100) | **100**  (94-100) | 98.3  (91.1-99.7) | **100**  (94-100) | **100**  (94-100) | 98.3  (91.1-99.7) |
| **PPV** (%) (95% CI) | NA | **100**  (20.7-100) | 0  (0-79.3) | NA | NA | 0  (0-79.3) |
| **NPV** (%) (95% CI) | 85.7  (75.7-92.1) | **87**  (77-93) | 85.5  (75.3-91.9) | 85.7  (75.7-92.1) | 85.7  (75.7-92.1) | 85.5  (75.3-91.9) |
| **Accuracy** (%) (95% CI) | 85.7  (75.7-92.1) | **87.1**  (77.3-93.1) | 84.3  (74-91) | 85.7  (75.7-92.1) | 85.7  (75.7-92.1) | 84.3  (74-91) |
| **Balanced Accuracy** (%) | 50  (50-50) | **55**  (50-66.7) | 49.2  (47.3-50) | 50  (50-50) | 50  (50-50) | 49.2  (47.4-50) |
| **F1 score** (95% CI) | NA | **18.2**  (12.5-54.5) | NA | NA | NA | NA |

*Prevalence of PVCs: 10/70* (14.3%)

CI, confidence interval; PPV, positive predictive value; NPV, negative predictive value.
NA: not applicable because no positives and/or no positive predictions.

**Table S10:** Diagnostic Metrics Regarding Identification of significant ST-Segment deviation

| **Metric** | **OpenAI**  **ChatGPT-4** | **OpenAI  ChatGPT-5** | **Google  Gemini-2.5** | **Microsoft Copilot** | **Claude  Sonnet-4** | **Claude  Opus-4.1** |
| --- | --- | --- | --- | --- | --- | --- |
| **Sensitivity** (%) (95% CI) | 7.1 (1.3-31.5) | 14.3  (4-39.9) | **21.4**  (7.6-47.6) | 0  (0-21.5) | 0  (0-21.5) | 0  (0-21.5) |
| **Specificity** (%) (95% CI) | 91.1 (80.7-96.1) | 98.2  (90.6-99.7) | 76.8  (64.2-85.9) | **100** (93.6-100) | **100**  (93.6-100) | 98.2  (90.6-99.7) |
| **PPV** (%) (95% CI) | 16.7  (3-56.4) | **66.7**  (20.8-93.9) | 18.8  (6.6-43) | NA | NA | 0  (0-79.3) |
| **NPV** (%) (95% CI) | 79.7  (68.3-87.7) | **82.1**  (71.3-89.4) | 79.6  (67.1-88.2) | 80  (69.2-87.7) | 80  (69.2-87.7) | 79.7  (68.8-87.5) |
| **Accuracy** (%) (95% CI) | 74.3  (63-83.1) | **81.4**  (70.8-88.8) | 65.7  (54-75.8) | 80  (69.2-87.7) | 80  (69.2-87.7) | 78.6  (67.6-86.6) |
| **Balanced Accuracy** (%) (95% CI) | 49.1  (42.6-58) | **56.2**  (48.3-66.5) | 49.1  (37.5-61.6) | 50  (50-50) | 50  (50-50) | 49.1  (47-50) |
| **F1 score** (95% CI) | 10  (7.7-30.8) | **23.5**  (10.5-50) | 20  (6.7-38.1) | NA | NA | NA |

*Prevalence of ischemia significant ST-Segment deviations:14/70* (20%)

CI, confidence interval; PPV, positive predictive value; NPV, negative predictive value.
NA: not applicable because no positives and/or no positive predictions.
*^a^* *κ*: agreement with human experts using Cohen’s kappa test.

**Table S11:** Comparison of Best Performing Generalist and Specialized LLMs Based on Balanced Accuracy Using an Identical Long Prompt.

| **Metric** | **Best** | **Best Generalist Balanced Accuracy  (95% CI)*** | **Best Specialized Balanced Accuracy (95% CI)*** | **Odds Ratio**  **(95% CI)******* | ***P* (McNe*mar Test)** |
| --- | --- | --- | --- | --- | --- |
| **Rhythm** | **PULSE-7B** | ChatGPT-4 55.8  (44.2-70.6) | PULSE-7B 86.3  (80.3-92.1) | 1.5 (0.6-3.7) | 1 |
| **First degree AV Block** | **ChatGPT-4** | Chat-GPT-4 57.8  (45.2-74.2) | PULSE-7B 48.8  (35.1-65.4) | 3.2 (1.2-8.7) | 1 |
| **Intraventricular Block** | **ChatGPT-5** | ChatGPT-5 67.8  (55.6-79) | PULSE-7B 64.9  (56-73.7) | 1.8 (0.9-3.6) | 1 |
| **IV-Block Type** | **ChatGPT-5** | ChatGPT-5 62.5  (50.2-74.5) | PULSE-7 62.6  (53.6-70.8) | 2.3 (1.1-4.6) | 1 |
| **QT-Prolongation** | **ChatGPT-4** | ChatGPT-4 61.3  (49.9-73.2) | PULSE-7B 47.9  (34.5-61.8) | 2.8 (1.1-7.2) | 1 |
| **PACs** | -^a^ | -^a^ | -^a^ | - | - |
| **PVCs** | **ECG-Instruct-Llama-3.2** | ChatGPT-5 55  (50-66.7) | ECG-Instruct-Llama-3.2 59.2  (42.1-76.2) | 4.8 (1.6-14) | 0.158 |
| **Ischemic ST-Changes** | **ChatGPT-5** | ChatGPT-5 56.2  (48.3-66.5) | ECG-Instruct-Llama-3.2 55.4  (45.8-63) | 4.1 (2.1-8) | **<.001** |
| **Axis Deviation** | **ChatGPT-5** | ChatGPT-5 71.3  (55.5-85.3) | PULSE-7B 70.3 (55-83.3) | 1.6 (0.7- 3.6) | 1 |

CI, confidence interval; AV, atrioventricular; IV, intraventricular; PACs, premature atrial contractions; PVCs, premature ventricular contractions

* 95% confidence intervals (CIs) are presented without adjustment for multiple comparisons. *P* values were adjusted using the Bonferroni method to control the family-wise error rate across all tests.

^a^ All models failed to correctly detect any PACs. Balanced accuracy of all models except ChatGPT-5 was 50 %, 95% CI (50-50), balanced accuracy of ChatGPT-5 was 49.3 %. 95% CI (47.7-50).

**Table S12:** Comparison of Best Performing Generalist and short-prompted Specialized LLMs Based on Balanced Accuracy

| **Metric** | **Best** | **Best Generalist Balanced Accuracy (95% CI)** | **Best Specialized Balanced Accuracy (95% CI)** | **Odds Ratio**  **(95% CI)** | ***P* (McNe*mar Test)** |
| --- | --- | --- | --- | --- | --- |
| **Rhythm** | **PULSE-7B (SP)** | ChatGPT-4 55.8  (44.2-70.6) | PULSE-7B (SP) 74.7  (59.3-90.1) | 0.4 (0.1-1.4) | 1 |
| **First degree AV Block** | **PULSE-7B (SP)** | ChatGPT-4 57.8  (45.2-74.2) | PULSE-7B (SP) 74  (59.1-84.4) | 2.7 (1.2-5.7) | .667 |
| **Intraventricular Block** | **ChatGPT-5** | ChatGPT-5 67.8  (55.6-79) | PULSE-7B (SP) 61.1  (49.8-71.6) | 1.9 (0.9-4) | 1 |
| **IV-Block Type** | **ChatGPT-5** | ChatGPT-5 62.5  (50.2-74.5) | PULSE-7B (SP) 57.5  (44.9-68.8) | 2.1 (1-4.5) | 1 |
| **QT-Prolongation** | **ChatGPT-4** | ChatGPT-4 61.3  (49.9-73.2) | PULSE-7B (SP) 57.8  (46.3-69.6) | 1.1 (0.5-2.3) | 1 |
| **PACs** | **PULSE-7B (SP)** | -^a^ | PULSE-7B (SP) 83.3  (50-100) | - | - |
| **PVCs** | **PULSE-7B (SP)** | ChatGPT-5 55  (50-66.7) | PULSE-7B (SP) 89.2  (74.2-100) | 0.25 (0.1-1.2) | 1 |
| **Ischemic ST-Changes** | **ChatGPT-5** | ChatGPT-5 56.2  (48.3-66.5) | PULSE-7B (SP)  53.6  (40.7-67.6) | 2.8 (2-8.5) | **.003** |
| **Axis Deviation** | **ECG-Instruct-Llama-3.2 (SP)** | ChatGPT-5 71.3  (55.5-85.3) | ECG-Instruct-Llama-3.2 (SP) 75.7  (61.1-90.2) | 0.4 (0.2-1) | 1 |

CI, confidence interval; SP, short-prompted; AV, atrioventricular; IV, intraventricular; PACs, premature atrial contractions; PVCs, premature ventricular contractions.

* 95% confidence intervals (CIs) are presented without adjustment for multiple comparisons. *P* values were adjusted using the Bonferroni method to control the family-wise error rate across all tests.

^a^ All generalist models failed to correctly detect any PACs. Balanced accuracy of all generalist models except ChatGPT-5 was 50%, 95% CI (50-50), balanced accuracy of ChatGPT-5 was 49.3 %. 95% CI (47.7-50).
